# Distinct coordination patterns integrate exploratory head movements with whole-body movement patterns during walking

**DOI:** 10.1038/s41598-022-26848-x

**Published:** 2023-01-22

**Authors:** Steven van Andel, Andreas R. Schmidt, Peter A. Federolf

**Affiliations:** 1 Department of Sport Science, University of Innsbruck, Fürstenweg 185, 6020 Innsbruck, Austria; 2 IJsselheem Foundation, Kampen, The Netherlands

**Keywords:** Human behaviour, Motor control

## Abstract

Visual guidance of gait is an important skill for everyday mobility. While this has often been studied using eye-tracking techniques, recent studies have shown that visual exploration involves more than just the eye; head movement and potentially the whole body is involved for successful visual exploration. This study aimed to assess coordinative patterns associated with head movement and it was hypothesized that these patterns would span across the body, rather than being localized. Twenty-one (after exclusions) healthy young adult volunteers followed a treadmill walking protocol designed to elicit different types of head movements (no stimuli compared to stimuli requiring horizontal, vertical, and mixed gaze shifts). Principal Component Analysis was used to establish whole-body correlated patterns of marker movement (Principal Movements; PMs) related to the activity of the head. In total 37 higher order PMs were found to be associated with head movement, two of these showed significant differences between trials associated with strong head rotations in the horizontal and sagittal plane. Both of these were associated with a whole-body pattern of activity. An analysis of the higher order components revealed that exploratory head movements are associated with distinct movement patterns, which span across the body. This shows that visual exploration can produce whole-body movement patterns that have a potentially destabilizing influence. These findings shed new light on established results in visual search research and hold relevance for fall and injury prevention.

## Introduction

Visual exploration is a fundamental behaviour that is crucial for the successful guidance of action in both humans^1,2^ and other animals^3–5^. In particular, visual exploration is crucial for the guidance of one of our most important behaviours: during locomotion, our visual system is used to safely navigate to targets while avoiding obstacles^6–9^. Perhaps, the significance of visual exploration does not become apparent in everyday walking, but this becomes more apparent when the perceptual-motor system is challenged to the point of failure (for example, try walking with your eyes closed), potentially leading to trips, falls or other injuries. As such, knowledge on the visual guidance of gait could be particularly relevant in these high-strain situations, which naturally occur in sport or situations that produce an elevated fall risk for older people. Interestingly, while the function of the visual system in isolation has been well studied^6^, how visual exploration behavior is coordinated with whole-body postural control is yet to be established.

The link between fall risk, eye movement and gait has been investigated previously^10^. Studies have emphasized the coordination between movements of the eyes and head during treadmill walking^11^, walking through different environments^12^ and when walking while searching^13^. Also, research exists that directly coupled characteristics of the gaze strategy to characteristics of gait, for example, Chapman and Hollands found that older people with an elevated fall risk needed more time to plan and execute mediolateral stepping adjustments^14^ and look away from stepping targets sooner than their low risk counterparts^15^, leading to a higher error in foot placement. Evidently, there is some change in the way we use visual exploration during gait that is associated with increasing fall risk.

The measurement of visual exploration in research is commonly operationalized using eye-tracking, a technique that records the focus of central vision. However, in everyday walking central vision alone does not cover the full function of visual exploration. That is, eye movements are often associated with head and body movement allowing for a greater range of exploration^10,12,13,16^. For example, research in association football has shown movements of the eyes as well as the head to be important for quantifying visual exploration^17^. The coordination between saccadic eye- and head movement is determined by the size of the gaze shift. Smaller gaze shifts are associated with only a saccade, with the head following behind to re-centre the eyes after the shift. Whereas gaze shifts that would orient the eyes near their mechanical boundary are made with a greater initial contribution of head movement^18^ or the entire body^19,20^.

Considering this coupling between eye, head and body movement, it is apparent that explorative activity could affect postural control. Since gaze shifts are often coordinated along with a delayed re-positioning of the head after a saccade^18^, we speculate that head movement activity might be delayed to a timing where they minimally affect the postural control of the whole body. Thus, if studying body movement patterns during walking while performing a visual exploration task, it could be expected that coordinative patterns emerge which integrate compensation strategies into the gait cycle.

Principal Component Analysis (PCA) is used for the operationalization of whole-body coordination assessments^21–23^. Using PCA, one can determine linear patterns or Principal Components that explain a part of the total variance in the dataset. These components represent movement strategies, i.e. patterns of correlated body segment movements, which have been referred to as ‘Principal Movements’ (PMs)^24^. Furthermore, PCA is a method of dimensionality reduction: the kinematic inputs with high dimensions can be reliably summarized in a small number of PMs. For example, during quiet stance or walking, the first three PMs already explain more than 90% of the variance^23,24^. Because of this aim in terms of data reduction, it is common to only look at the first couple of PMs until some criteria is satisfied. This procedure is implemented, reasoning that the higher order components explain only a small portion of the total variance per component and have a worse signal to noise ratio. However, recent findings suggest that in human movement data, these higher components might still contain relevant information, reflecting smaller, more localized movements or faster control strategies^25,26^ or movement patterns associated with a small amplitude like breathing or head movement in standing balance^24^. In gait, head movements are expected to be among the higher order PMs, since the lower order PMs will likely be overshadowed by the bigger amplitude of arm and leg movements, and thus we reason that a focus on higher order movement components is warranted^26^.

The aims of the current study are to identify specific movement coordination patterns that can be associated with exploration-induced head movements during walking. Contrasting expectations are possible about these specific patterns. Firstly, these patterns could be independent from the gait pattern, resulting in the identification of head movement PMs that see very little representation of other body movement. However, we hypothesize that head movement would be related to whole-body movement. In this case, PMs that represent head movements will also encompass distributed patterns. An analysis of these distributed patterns should provide insight into the compensation strategies employed to assist in moving the head while walking.

## Results

### Head movement

**Figure 1:**
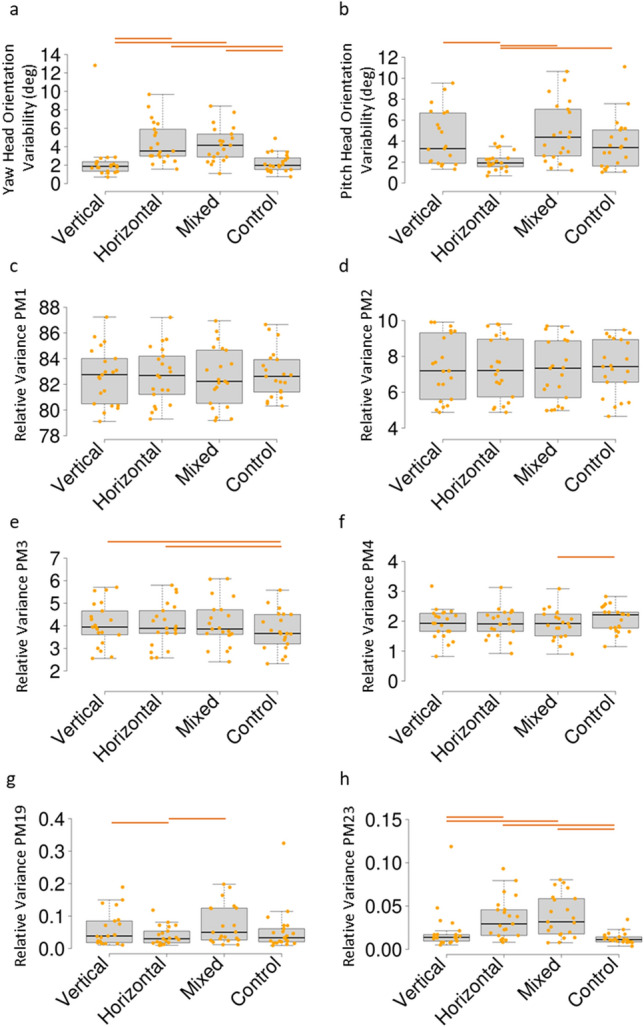
Boxplots of between condition effects of head movement variability (**a** and **b**), the main movement patterns identified by PCA (PM1–PM4, panel **c–f**) and the two PMs mainly associated with head movements (PM19 and PM23, panel **g** and **h**). Horizontal bars above panels indicate significant differences using pairwise t-testing (panel **a** and **b**) and pairwise Durbin-Conover tests (panel **c**–**h**), all with Bonferroni correction for multiple comparisons.

The visual exploration stimulus implemented during treadmill walking was successful in eliciting head movement, as shown by a significant effect of stimulus orientation (‘condition’) on the orientation of the head markers (Fig. 1a,b; head pitch: F(3,54) = 10.5, *p* < 0.001, η^2^ = 0.170; head yaw: F(1.23, 22.09) = 24.8, *p* < 0.001, η^2^ = 0.369). This is an important assumption in the analysis, since the distance between the LEDs that were used as stimuli technically do not require head movement from a participant (that is, a saccade might suffice).Bonferroni corrected (α = 0.05/6 = 0.008) pairwise comparisons in the head pitch angle variation showed significant differences between the horizontal condition and all other conditions (all *p*-values < 0,008) but not among any of the other conditions. For the head yaw angle variation, Bonferroni-corrected pairwise comparisons identified significant differences between the horizontal condition and both the vertical and control conditions (*p*-values < 0.008) and between the mixed condition and the vertical and control conditions (*p*-values < 0.008); differences were not significant between the vertical and control and horizontal and mixed pairs. Note: Shapiro-Wilk tests had supported a normality assumption, however, the analysis identified two outliers more than 3.5 standard deviations removed from their group averages, which were excluded from the head movement analysis (Fig. 1a,b).

### Head-body coordination

**Figure 2:**
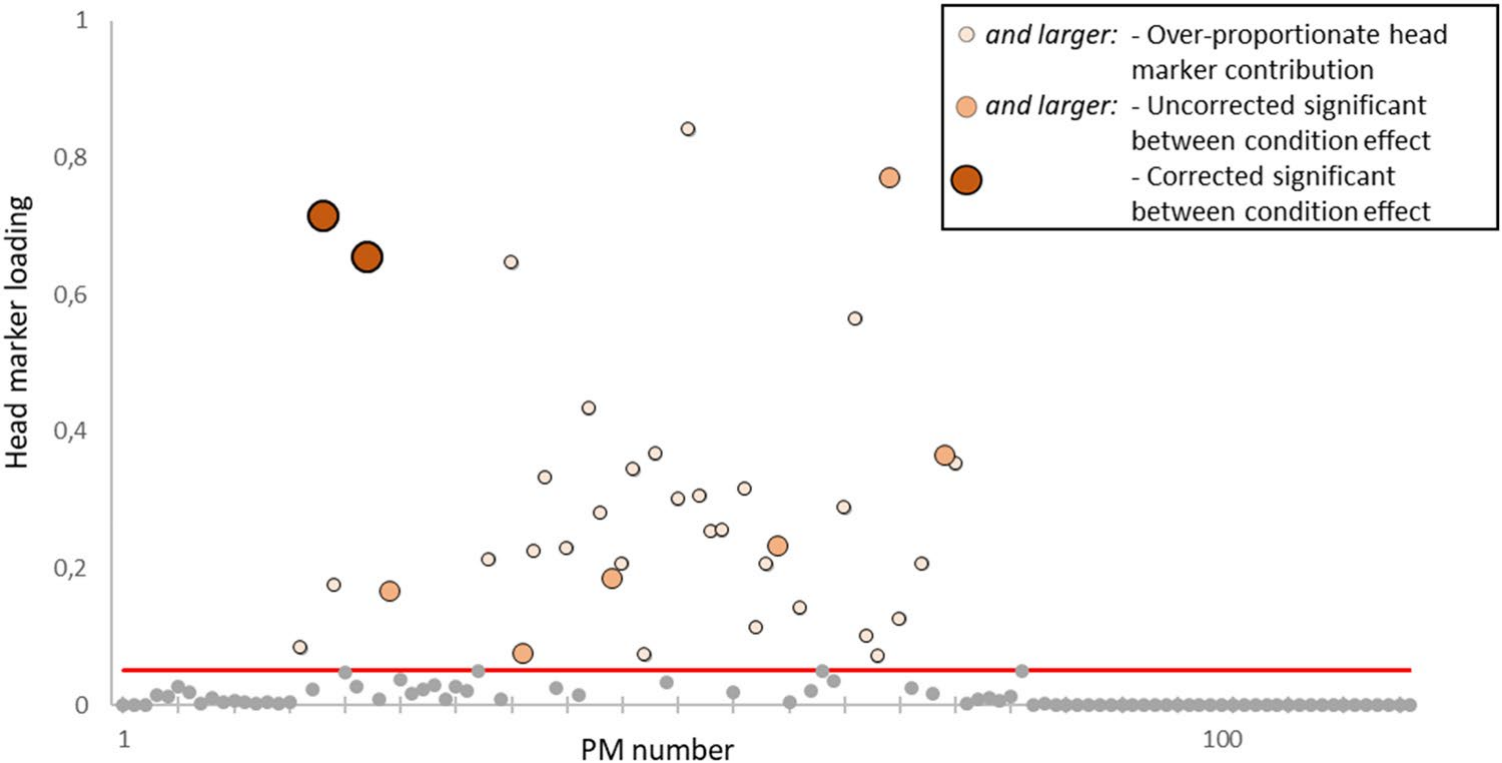
Sum of the head-marker loading per PM (range: PM1–PM117). The horizontal line represents the threshold value of 5% (approximate body mass represented by the head), indicating that loadings above this line have relatively high head activity. Thirty-seven PCs were found with a combined head marker loading above the threshold. Shading and size of markers above threshold indicates between-condition differences in a Friedman test, where the smallest markers (light shade) show no difference, the medium sized markers (medium shade) show a difference with an uncorrected alpha of 0.05 and the largest (darkest) markers show significant differences even with an alpha corrected for 37 parallel comparisons. It is important to note here that in all PMs that show between condition effects, head movement is not isolated but at least 20% of variance is found in distributed patterns.

Two PMs were required to explain about 90% of the variance in the dataset and five PMs were present with a relative eigenvalue greater than 1 percent (supplementary Fig. 1). Differences between conditions in these lower order PMs are illustrated in Fig. 1c–f. Figure 2 shows the sum of the head marker loadings in each PM. The following trends in Fig. 2 are noteworthy: 1) No over-proportionate (that is; a PM with a head marker loading greater than the approximate weight-percentage represented by the head in a average body) head marker contributions were found among the first 16 PMs. 2) a first cluster of head marker loadings are found around PM17-25. 3) a second, bigger cluster can be found between PM34 and PM76. 4) no over-proportionate head marker contributions are found between PM77 and 117. In total, 37 PMs were identified with an over-proportionate head marker loading (contribution greater than 5%), which were analysed further in terms of the differences in relative variance between conditions.

### Relative variance

**Figure 3:**
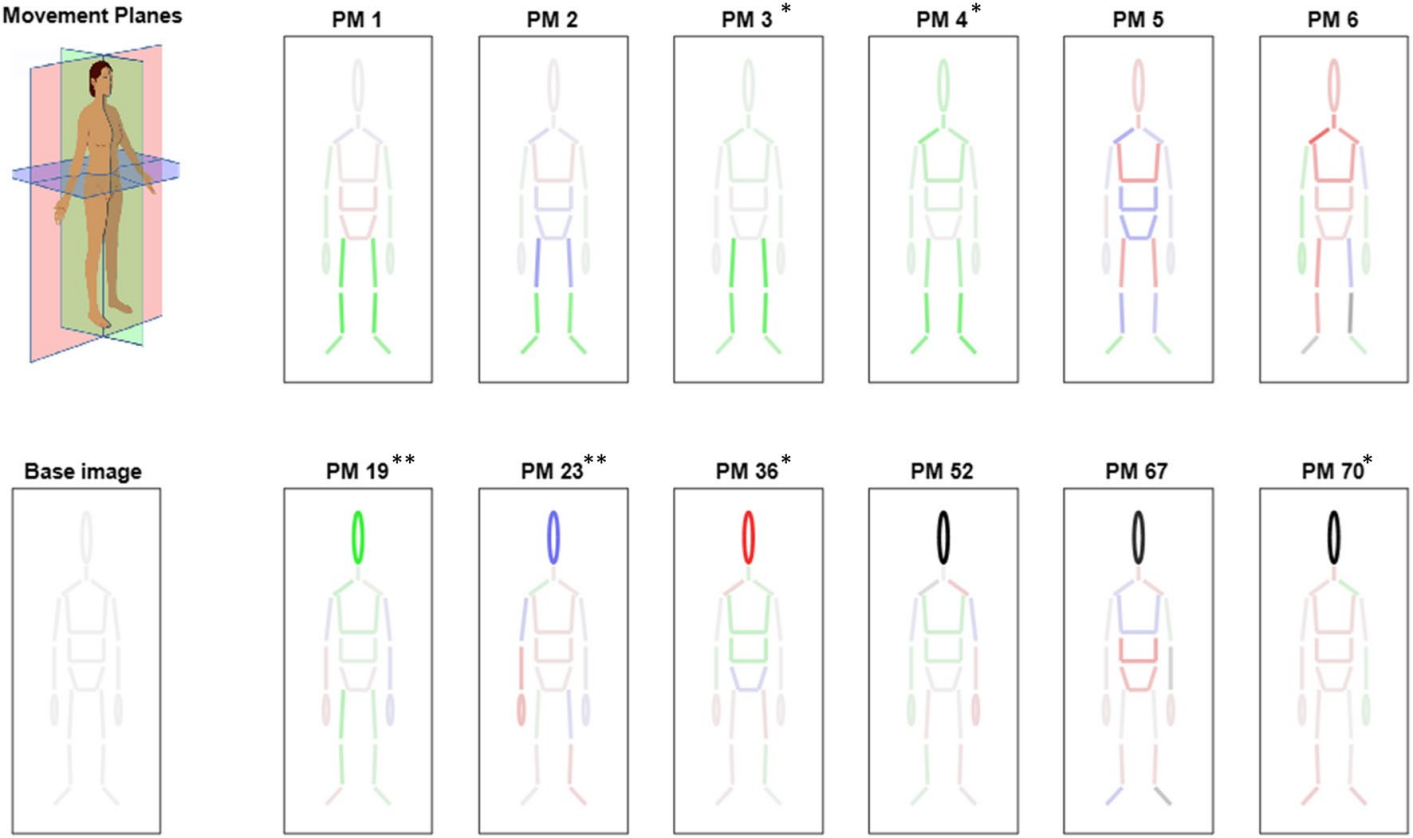
Visual representation of marker loading per body segment for the first 6 PMs and the 6 with the highest head marker loading. Movement Planes panel shows a colour index for the other panels: body segments depicted green are most active in the sagittal plane, segments depicted red are most active in the frontal plane and segments depicted in blue are most active in the horizontal plane. Movement Planes panel images is adapted from: https://commons.wikimedia.org/wiki/File:Planes_of_Body_unlabeled.jpg, under CC4.0, using paint.net. ‘Most active’ in this case means that the segment loading reached over 80% contribution on the two axes within a plane (e.g. if the contribution of the anterior-posterior and the vertical segment loading together reached over 80%, contribution is classified as sagittal plane activity). Body segments in greyscale are not dominant in any specific plane. Base image panel shows the image in case all marker loadings would equal 0 to set a baseline for the PM panels. PM panels show marker loadings per body part, darker shades indicate greater marker loading for this segment. Asterisks indicate significant differences between conditions, where a single asterisk indicates significance in an uncorrected Friedman test and a double asterisk indicates significance in a Friedman corrected for 37 parallel comparisons. Of note here are the patterns associated with head movements which appear distributed across the body.

The relative variance from the 37 head-movement PMs were subjected to a Friedman test to assess differences between different stimulus orientations. Due to the increased type I error associated with 37 parallel comparisons, a Bonferroni correction was applied (α_c_ = 0.05/37 = 0.0014). However, considering the increased type II error with such a strong alpha correction, uncorrected values were also reported. Eight head-movement related PMs showed significant differences between conditions with an uncorrected alpha (medium markers in Fig. 2: PM19, PM23, PM25, PM37, PM45, PM60, PM70, PM75). Two PMs were identified to show significant differences between conditions after alpha correction (Fig. 1g,h and large markers in Fig. 2;). Firstly: PM19 (χ2(3) = 19.00, *p* < 0.001), Durbin-Conover pairwise comparisons (Fig. 1g) with Bonferroni correction (α_c_ = 0.008) revealed significant differences between the horizontal and the vertical condition (*p* < 0.001), as well as the horizontal and mixed condition (*p* < 0.001). Secondly, significant results were found in PM23 (Fig. 1h, χ2(3) = 36.50, *p* < 0.001). Bonferroni-corrected (α_c_ = 0.008) Durbin-Conover tests showed significant effects between the horizontal and both the vertical and the control conditions as well as between the mixed and both the vertical and the control conditions. PM19 describes correlated marker trajectories related to a strong sagittal plane rotation of the head (i.e. pitch) with distributed patterns in the arms and legs, also mostly in the sagittal plane (Fig. 3). PM23 describes a strong horizontal plane head rotation (i.e. yaw), along with distributed frontal plane patterns in the left arm and the trunk and mixed activity in the legs (Fig. 3).

## Discussion

Results confirmed that the gaze direction task induced head rotations, which were associated with coordinative patterns that span across the body. Head movements in the sagittal plane were associated with a distributed sagittal plane movement pattern in the torso and legs, while head movements that occurred in the horizontal plane were mainly associated with frontal plane movement patterns in the left arm and right lower leg. It should be noted that the outcomes of the PCA analysis are specific to this experimental paradigm and replication in natural walking (under a set of very different constraints^27,28^), where exploration is often self-paced rather than prescriptive, will result in different PCA-characteristics such as head movement PM orders and segment loadings. The relevant take-home messages therefore do not lie in these details of the PMs, but rather in the general pattern of the findings. The main messages are: 1. exploratory head movements are associated with distributed movement patterns across the body. 2. These movement patterns were not represented in the lower-order components, but higher order PMs need to be assessed to understand the coordination between eye, head and body. 3. Sagittal plane head movements were generally associated with sagittal plane body segment activity while horizontal plane head rotations were more associated with frontal plane activity.

n a functional level, these results are not surprising. Two possible patterns of activity would have been possible to accommodate for the visual exploration task. Firstly, a head-localized strategy would be possible in which head movements are largely independent from body movements. However, such a strategy would require more extreme neck movements and a strong, inefficient, fixation of the body. As the task of walking is relatively simple, such a ‘freezing’ strategy^29^ is not ideal and as hypothesized, the current results established whole-body coordinative patterns to accommodate visual exploration. In this low-demand environment, the need to stabilize and to explore can coexist: postural movement can be allowed, and they do not lead to events of instability. However, it could be a future hypothesis that in situations where balance is challenged, such exploratory fluctuations might lead to instability, indicating a trade-off between stability and exploration. During walking people will usually be well able to explore visually before actively engaging the entire body^30^. Here, we have shown that even when walking under low demands, exploratory head movement are still associated with whole-body coordination during gait, implying that an exploration-vs-stabilization trade-off might exists during gait. Where people find themselves in this trade-off would be a result from the constraints involved in the execution of the task^27^. That is, when a task is simple and/or an individual is very skilled (as was the case in the current study), then the trade-off is not stressed and active exploration becomes an opportunity for action^31^. However, when stability demands rise, less exploration will be afforded. It is a recommendation for future studies to further establish this trade-off and how people response to high demands to stability as well as exploration.

A potential field where insights into the whole-body compensation strategies associated with visual exploration might be relevant is in sports. Here, situations occur where both exploratory and stability demands are high. A postural compensation strategy related to visual exploration, in addition to the already high postural demands of sport (e.g. handling equipment while sprinting, jumping or sharply turning), might increase the risk for injuries. Although speculative at this point the exploration-vs-stabilization trade-off described here might prove valuable in explaining injuries such as anterior cruciate ligament injuries that are known to occur often when athletes find themselves near an opponent (indicating increased exploratory demands), when balance is perturbed or when engaging in a challenging manoeuvre (indicating increased stability demands) such as decelerating or side-cutting^32^. There is an opportunity for future studies here to quantify the exploratory and stability demands in the instances leading up to injury events using video analysis. The distributed coordinative patterns associated with exploration might prove to play a disrupting role in the cause of injury.

While many studies use PCA as a method for dimensionality reduction and aim to only analyse a low number of components while retaining the critical information, we reported results that clearly indicated potentially relevant information could still be present in the higher order components. If the current study would have followed traditional guidelines and would have only included PMs until a ‘marked drop’ in eigenvalues can be observed^22^, until we would have reached 90% of explained variance e.g.^33–35^ or by including all PMs that explain more than one percent of the total variance^36^, then we would have executed an analysis on respectively one, two or five PMs (Supplementary Fig. 1). In terms of head movements, this would have limited the analysis to a focus on PM3 and PM4, which showed differences between conditions (Fig. 1e,f), but would have excluded the PMs that showed patterns resembling rotations of the head: PM19 and PM23 (compare Fig. 1a,b,g,h). This analysis proves that while a focus on the lower order PMs might be a good strategy for dimensionality reduction, a focus on the higher order PMs enables an analysis of specific movement patterns that occur systematically throughout the movement.

In summary, the current study assessed whole body coordinative patterns associated with visual exploration induced head movements during gait. Analysing the higher order components resulting from PCA, we established that exploratory head movements are associated with specific movement patterns across the body, where sagittal plane head rotations were generally associated with sagittal plane activity and transverse plane head movements were more associated with frontal plane activity. These results imply an exploration-vs-stabilization trade-off that could hold relevance for better understanding visual exploration during gait, as well as injuries occurring during gait. Further research should assess the generalizability and applicability of results towards everyday gait in the general population, as the investigated relationships hold relevance for balance and postural control research across the lifespan.

## Methods

### Participants

A sample of 23 participants (13 women, mean age: 25.7) were recruited from the local student body, however, two participants were excluded due to measurement errors after visual data inspection. All participants were free of lower extremity injury in the past 6 months or any other health problems that could potentially affect their gait pattern. The study’s protocol was approved by the Board for Ethical Questions in Science of the University of Innsbruck (38/2020) and executed in accordance with the Declaration of Helsinki. All participants provided informed consent before participating in the study.

### Procedure

**Figure 4:**
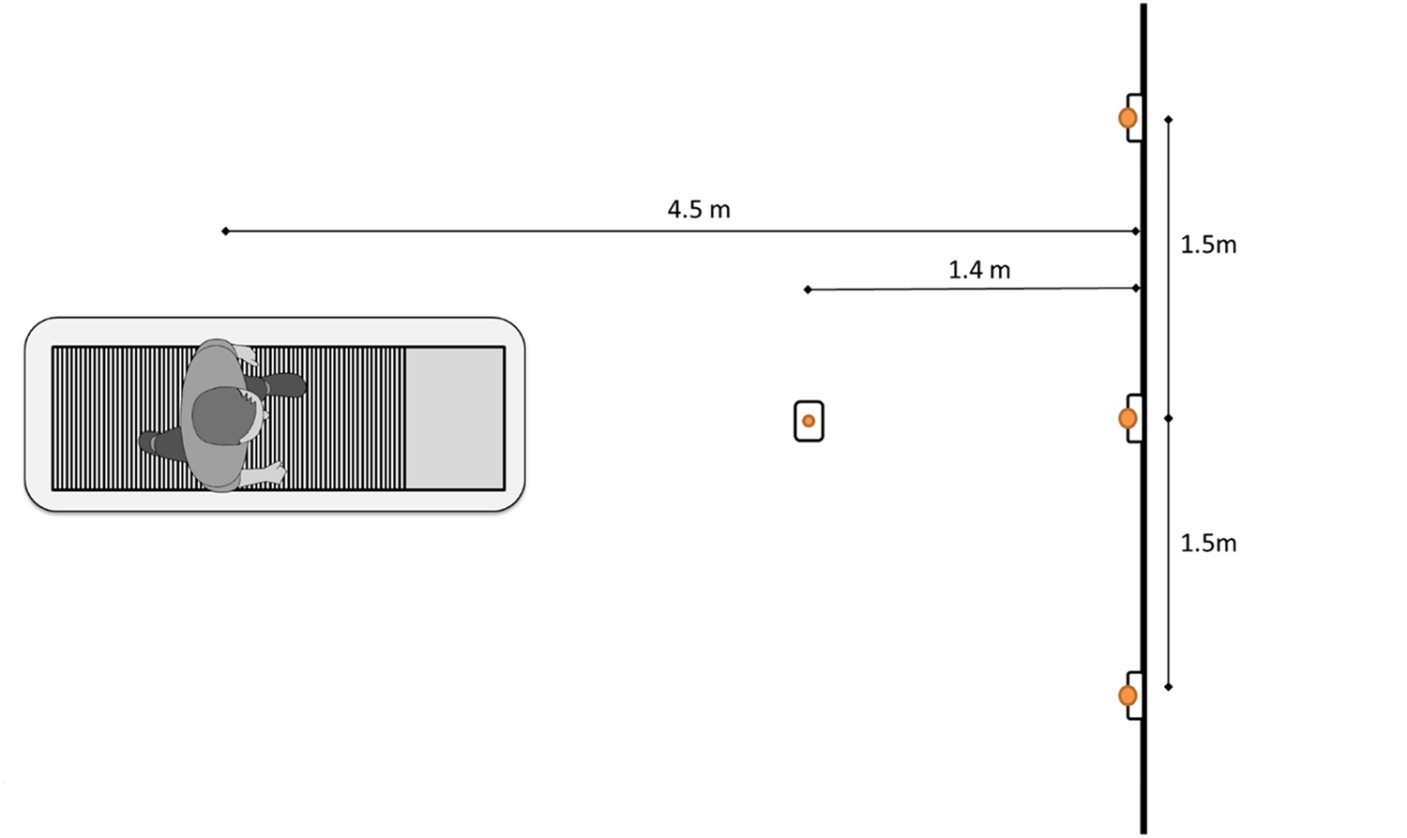
Top view of experimental set up. Three right LEDs are mounted on a wall at approximate eye-height from the treadmill. The LED positioned between the treadmill and the wall was positioned on the ground, at a distance that represents approximately 2 steps ahead from the centre of the treadmill. Horizontal plane head rotation (yaw) required for a movement between the most lateral LEDS is about 37°. Sagittal plane rotation required from the ground LED to the wall mounted LEDs is about 49°. Exact angles depend on participant height and precise position on treadmill.

Participants were equipped with 39 reflective markers (Vicon full-body plug-in gait marker set). Ten infra-red cameras were used to record marker locations at 250 Hz (Vicon Motion Systems Ltd, UK). A treadmill was positioned in the centre of the lab and the speed of the treadmill was set to 4 km/h for all conditions to make the resulting data comparable between participants. To stimulate eye and head movement, a programmable set of LED lights were used. Three LEDs were fixed on approximate eye-height on a wall in front of the participant and one was fixed on the ground between the treadmill and the wall (Fig. 4). The LEDs were programmed to light up one at a time and the participant was instructed to follow this lighting-up sequence with their gaze.

Participants stepped onto the treadmill and first completed a ‘control’ measure by walking without specific instruction for 2 min. After a short break, the participant got back on the treadmill and the LED-sequence was activated. The LEDs lighted up in automated order over 6 min in different settings as specified in Table 1. Different frequencies were introduced to ensure that participants could not synchronize their exploration to their gait cycle or vice versa, but since this was not the main aim of the study, averages between low and high frequency recordings were used in the further analysis (i.e. one value for vertical, horizontal, mixed and control conditions).

**Table 1:** Overview of experimental conditions.

Time (minute)	LEDs involved; stimulated head movement	Frequency
0–2	Control; No stimulated head movement	
0–1	Central LEDs; Vertical head movement	Low^A^
1–2	Central LEDs; Vertical head movement	High^B^
2–3	Lateral LEDs; Horizontal head movement	Low^A^
3–4	Lateral LEDs; Horizontal head movement	High^B^
4–5	All LEDs; Mixed head movement	Low^A^
5–6	All LEDs; Mixed head movement	High^B^

^A^LED change unpredictably every 3.5–4.5 s.

^B^LED change unpredictably every 2–3 s.

### Data analysis

Recorded marker trajectories were reconstructed and labelled using Vicon Nexus (version 2.9.2) and then exported for further processing in MATLAB (MathWorks Inc., Natick, MA, USA). Using MATLAB, datafiles (rows: timepoints, columns: marker trajectories) were cut to 50-s files containing steady-state walking in each condition (cutting off 5 s before and after the change of condition to avoid potential synchronization and settle-in issues). Then, the origin of the axes system was reset to be central between the posterior left and right iliac spine markers to minimize the influence of any position shifts on the treadmill on the results from the analyses.

#### Head movement analysis

To assess the effectiveness of the manipulation, head orientation was computed using the four head markers. This resulted in two variables describing the head rotation in the sagittal (pitch) and horizontal plane (yaw). Standard deviations of these trajectories were determined to give a measure of the amount of rotation in each plane over a complete trial. Outliers were removed if they were more than 3.5 SD distant to the group mean and normality was assessed using Shapiro-Wilk tests. A repeated measures analysis of variance (RM-ANOVA) with Bonferroni-corrected pairwise comparison was used to assess condition effects on the standard deviation of the pitch and yaw trajectories (representing the amount of variation in the sagittal and horizontal plane, i.e. activity in each rotation). In case of violations to the assumption of sphericity, a Greenhouse-Geisser correction was applied. The analysis was performed in Jamovi (version: 2.2.5) with alpha set to 0.05.

#### Principal component analysis

Datafiles were further processed using the ‘PManalyser’, an open-source MATLAB-based software package for PCA^37^. The following pre-processing steps were completed: 1) Implement a ‘Mean Euclidian Distance’ normalization^24,37^ that firstly redefines recorded postures in terms of their deviation from the mean posture for each trial and secondly rescales variance of the individual datafiles so all datafiles have an equal influence on the resulting analysis. Together, this minimizes the influence of anthropometric differences between participants (i.e. a large person taking bigger steps affects the analysis equal to a small person taking smaller steps). 2) For each marker trajectory, a weighing was applied in terms of the percentage body weight represented in each body segment (based on a combination of data from^38–40^). The weighting of the asymmetrical markers was set to a value close to zero to minimize their influence on the analysis. The marker weighting was implemented to achieve a better balance between any small amplitude, whole-body compensation strategies and large movements shown in lighter segments such as the hands. 3) A data matrix is computed from all separate datafiles concatenated vertically so that different marker trajectories are represented in columns and different participants, conditions and frames are all represented in a nested structure in the rows. Thanks to these normalization and concatenation steps, one PCA could be performed on the entire dataset from all participants together and results become directly comparable between participants^24^.

### Head-body coordination

PCA results in a set of PM eigenvalues, and the relative variance explained by each component. To quantify the engagement of specific body segments in specific PMs, the loading scores of each marker onto each PM were computed. Loading scores were summed together for markers placed on one body segment (totalling at 19 segments: the head, neck, chest, abdomen, and pelvis, and two shoulders, upper arms, forearms, hands, upper legs, shanks and feet) to assess the contribution of each segment in the specific PM. PMs where the head markers provided a relatively high loading were considered for further analyses. The threshold for this was set at a minimum loading of 5% from the head markers, as the percentage of body weight represented by the head is about 5%^38–40^.

### Relative variance

For those PMs that were identified as having a major head movement contribution, the relative variance output was further analysed. Changes in relative variance per PM between conditions reflect changes in the amount that a recorded PM contributed to the overall body movements during each condition. A significant change therefore indicates that the overall movement structure changed. Due to issues with the normality of these variables, Friedman tests were employed to assess differences between conditions in relative variance for all head-movement related PMs. Alpha was set to 0.05, however a Bonferroni correction was applied to the outcomes to mitigate the risks of alpha inflation. Both corrected and uncorrected outcomes are reported to provide an objective overview of the analysed effects.

## Supplementary Information

Supplementary Information.

## Data Availability

The datasets used and/or analysed during the current study available from the corresponding author on reasonable request.
