# Supplementary material for: Distinct coordination patterns integrate exploratory head movements with whole-body movement patterns during walking
Source: Sci Rep. 2023 Jan 22;13:1235. doi: 10.1038/s41598-022-26848-x (PMC9868120; doi:10.1038/s41598-022-26848-x)
Supplement: Supplementary file 1 — Supplementary Information. [file 41598_2022_26848_MOESM1_ESM.docx]

**
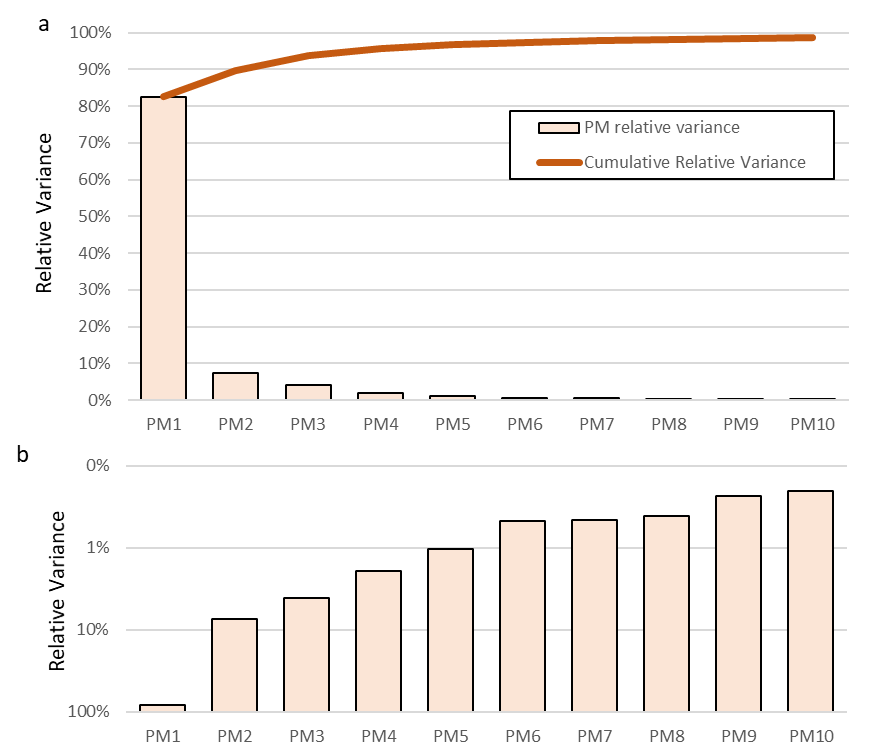
**

**Supplementary Figure 1. Explained variance per Principal Component. a) shows the variance explained by each individual component (bars) and the cumulative variance computed by counting up the bar values (line). b) logarithmic transform of the bars depicted in (a), magnifying the differences between higher order components.**
